# Glutamine synthetase mutations that cause glutamine deficiency: mechanistic insights from molecular dynamics simulations

**DOI:** 10.1186/2047-783X-19-S1-S15

**Published:** 2014-06-19

**Authors:** Benedikt Frieg, Nadine Homeyer, Dieter Häussinger, Holger Gohlke

**Affiliations:** 1 Institute for Pharmaceutical and Medicinal Chemistry, Heinrich Heine University, 40225 Düsseldorf, Germany; 2 Clinic of Gastroenterology, Hepatology and Infectious Diseases, Heinrich Heine University, 40225 Düsseldorf, Germany

## 

Glutamine synthetase (GS) is a key enzyme in nitrogen storage and metabolism as it catalyzes the ligation of glutamate and ammonia to glutamine with the help of ATP [1]. The specific function of GS depends on its localization: In astrocytes in brain tissue, GS is part of the glutamate-glutamine cycling, that way detoxifying cytotoxic ammonia and neurotoxic glutamate by conversion to glutamine; several links between the loss of GS activity and neurological disorders such as Alzheimer’s disease and epilepsy have been described. In liver tissue, GS plays an important role in eliminating ammonia; a loss of GS activity there leads to hyperammonemia, the main trigger of hepatic encephalopathy. Two mutations in GS (R324C and R341C) have been linked to congenital glutamine deficiency with severe brain malformations resulting in neonatal death [2]. In a single case known to date, another GS mutation (R324S) was identified in a boy, now five years old, who is neurologically compromised [3]. So far, the molecular mechanisms of these mutations on GS deactivation have not been understood.

**Figure 1:**
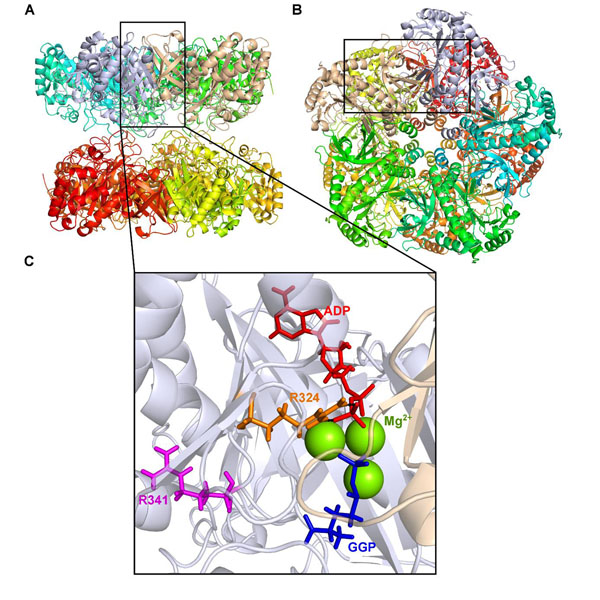
Structure of human GS (PDB entry: 2QC8 [1]) in cartoon representation; (A) top view, (B) side view. Each subunit is colored differently. (C) Close up view of the active site of human GS with residues R324 (orange) and R341 (magenta), ADP (red), and GGP (blue) shown in sticks representation and three Mg^2+^ ions depicted as green spheres. The salt bridge between R324 and ADP is shown as a black dotted line.

We performed molecular dynamics (MD) simulations of human wild type GS (wtGS) and the GS mutants R324C, R324S, and R341C in order to reveal the molecular mechanisms of GS deactivation. For the wtGS and the three mutants, four different states each of the enzymatic process were simulated for a length of 100 ns, resulting in a total simulation time of 1.6 µs for systems of ~35,000 atoms. In mammals GS is a homodecamer formed by two pentameric rings, with the active sites located at the interfaces between two dimers (Figure 1A, B). To reduce computational costs, we performed the MD simulations on dimers of GS only. Initial tests on wtGS showed that this leads to similar structural and dynamics features in the active site as observed for the decamer.

The MD simulations revealed for each GS mutation a distinct influence on GS’s highly regulated catalytic mechanism [4]. According to this mechanism, upon binding of the co-factor ATP the flexibility of a loop region, referred to as glutamate flap [4], increases, which enables binding of glutamate. Next, the terminal phosphate group of ATP is transferred to the glutamate forming ADP and the intermediate γ-glutamylphosphate (GGP). In this state, the flap loses its flexibility and seals the active site. This prevents water from entering and, thus, an early hydrolysis of GGP, which would lead to a nonproductive catalytic cycle otherwise. The flap behavior observed in our MD simulations of wtGS agrees with this mechanism.

The side chain of R324 in wtGS stabilizes in the GGP bound state the position of the terminal phosphate group of ADP by a salt bridge (Figure 1C). In contrast, in mutant R324C, the flap region is highly flexible in all catalytic stages, leading to a widely open glutamate binding site even after GGP has been formed, which may result in an early hydrolysis of GGP. The R324C mutation also leads to a loss of the direct interaction with ADP, resulting in a higher mobility of the ligand in the binding pocket. Both effects can explain the observed loss of GS activity in the R324C mutant [2]. While the R324S mutation also leads to a loss of the direct salt bridge interaction with ADP, this interaction is partially restored by bridging water molecules in between the sidechain of 324S and the ligand. This indirect, water-mediated interaction yields a partial stabilization of the ligand. Nevertheless, the flap’s flexibility in this state is still higher than in wtGS. As a result, the ligand is more mobile than in wtGS but not as mobile as in the R324C mutant. This can explain why still a residual activity was observed in the R324S mutant [3].

R341 in wtGS is not part of the catalytic site but is located close to the surface of GS where it connects two topologically separated regions. The analysis of the mechanical stability of these regions by CNAnalysis (http://www.cnanalysis.de) identified R341 as an import stabilizing residue. Accordingly, structural distortions in the neighborhood of the mutation site were observed in the course of the MD simulations for the R341C mutant. These structural changes on the surface of GS may explain the observed loss in affinity for antibody binding to this mutant [2]. The R341C mutation also exerts a long-range effect on the GS active site in that the flap loses its flexibility after ATP binding. This is expected to hamper the ATP-dependent glutamate binding because the glutamate binding site is not able to open anymore, which can explain the observed loss of GS activity in the R341C mutant [2].

In summary, our MD simulations provide detailed insights into the molecular mechanisms of GS deactivation by three clinically relevant mutations. The R324C and R341C mutations exert direct or long-range structural effects, respectively, that are associated with a complete loss of GS activity. In contrast, the effect of exchanging R324 with serine is partially compensated by water-mediated interactions, resulting in a residual activity of GS. To date no adequate treatment of a glutamine deficiency due to GS deactivation is available [3]. Our results allow suggestions for how to counteract the mutation effects, in particular in the case of the mutant R324S. On the one hand, one can aim at overall stabilizing the mutant protein by changes in the solvent composition adding compatible solutes. On the other hand, one can aim at restoring original interaction strengths between a mutated active site residue and ligands of GS either by noncovalently bound adaptor molecules or by covalently attached grafting molecules. The latter will result in chemical protein repair.
